# Metalloporphyrins as Tools for Deciphering the Role of Heme Oxygenase in Renal Immune Injury

**DOI:** 10.3390/ijms24076815

**Published:** 2023-04-06

**Authors:** Elias A. Lianos, Maria G. Detsika

**Affiliations:** 1 Veterans Affairs Medical Center and Virginia Tech, Carilion School of Medicine, Salem, VA 24153, USA; 2 GP Livanos and M Simou Laboratories, Evangelismos Hospital, 1st Department of Critical Care Medicine & Pulmonary Services, National and Kapodistrian University of Athens, 10675 Athens, Greece

**Keywords:** heme oxygenase, immune mediated, renal injury

## Abstract

Renal immune injury is a frequent cause of end-stage renal disease, and, despite the progress made in understanding underlying pathogenetic mechanisms, current treatments to preserve renal function continue to be based mainly on systemic immunosuppression. Small molecules, naturally occurring biologic agents, show considerable promise in acting as disease modifiers and may provide novel therapeutic leads. Certain naturally occurring or synthetic Metalloporphyrins (Mps) can act as disease modifiers by increasing heme oxygenase (HO) enzymatic activity and/or synthesis of the inducible HO isoform (HO-1). Depending on the metal moiety of the Mp employed, these effects may occur in tandem or can be discordant (increased HO-1 synthesis but inhibition of enzyme activity). This review discusses effects of Mps, with varying redox-active transitional metals and cyclic porphyrin cores, on mechanisms underlying pathogenesis and outcomes of renal immune injury.

## 1. Introduction

Renal immune injury is a frequent cause of end-stage renal disease (ESRD) in patients entering long-term dialysis and kidney transplantation programs worldwide. Immune injury frequently targets the renal glomerular microvasculature (glomeruli), which is highly vulnerable as many endogenous or exogenous antigens have the potential to either directly deposit in glomeruli or form immune complexes initiating an inflammatory response (glomerulonephritis, GN) [1]. Studies with animal models of GN confirmed the crucial interactions between blood-borne inflammatory cells infiltrating glomeruli and intrinsic glomerular cells. Understanding these interactions has enabled the elucidation of various aspects of GN and allowed major advances in pharmacologic approaches for renal function preservation. However, GN treatment is still lagging. Although interventions including administration of antibody-based treatments (anti-CD3, anti-CD20, anti-complement component proteins), inhibition of antigen-specific auto-reactive effector T cells, steroids, cytotoxic therapies, and plasma exchange can result in satisfactory renal function outcomes when initiated early, they may also cause general immunosuppression and lead to an increased risk for opportunistic infections and malignancy. Hence, the need for innovative and novel therapeutic approaches, such as use of molecule-specific disease-modifying agents, remains crucial. The cytoprotective enzyme heme oxygenase (HO) has emerged as a promising molecule amenable to modulation by specific naturally occurring or synthetic substrates referred to as Metalloporphyrins (Mps). This review discusses the role of Mps in modulating underlying mechanisms of renal immune injury and their potential as disease modifiers.

## 2. Effector Mechanisms in Renal Immune Injury

### 2.1. Complement Activation

A histopathologic examination of kidney lesions in immune-mediated injury, such as GN or interstitial nephritis (IN), invariably reveals antibody and complement depositions, the presence of leukocytes, and, occasionally, coagulation. Antibody deposition within glomeruli is frequently the initiating event of injury in several forms of human and experimentally induced GN. Furthermore, it may lead to inflammation as a result of antibody-dependent cell-mediated cytotoxicity via Fc receptors present on intrinsic kidney cells or by antibody-mediated activation of the complement cascade [2,3,4]. Complement activation within glomeruli is a key feature in many forms of GN, and a pathogenetic role has been firmly established [5]. In addition to initial injury, complement activation has been implicated in driving kidney damage progression [6]. The important role of complement as an effector mechanism of kidney damage has been confirmed by promising outcomes of complement inhibitory antibody treatment strategies, such as anti-C5 antibody therapies, in experimental and human GN [7]. The blood coagulation cascade is also activated within nephritic glomeruli and is triggered primarily by tissue factor (also known as platelet tissue factor, factor III, or CD142). It results in intraglomerular thrombin formation and deposition with pro-inflammatory effects and hemodynamic consequences [8,9].

### 2.2. Leukocyte Recruitment and Infiltration

A consistent histopathological feature of GN and IN is the presence of leukocytes within glomeruli and the interstitium. The rate of proinflammatory leukocyte accumulation was shown to correlate with clinical and pathological severity, as well as disease outcomes in both animal and human forms of GN. Leukocytes accumulate as a result of recruitment from the circulation and subsequent proliferation within the kidney. Recruitment from circulation is directed by chemokines generated within the kidney following the onset of antibody or complement-mediated injury [10]. The mechanisms by which the recruited leukocytes cause injury include the release of proinflammatory cytokines, cell death via the release of reactive oxygen and nitrogen species, and the release of metalloproteinases which degrade the extracellular matrix and cause tissue remodeling [11]. Most inflammatory leukocytes found in various forms of human and experimental GN are macrophages and play an important role in recruiting cytotoxic CD8^+^ lymphocytes [2] and regulating CD8^+^ T cell-dependent injury [3]. Infiltrating macrophages were shown to be bone marrow-derived and are programmed by specific cytokines to either increase intensity of the inflammation or promote its resolution [4]. An established pattern of macrophage activation involves augmented inducible nitric oxide synthase (iNOS)-derived nitric oxide (NO) [12], and NAD(P)H oxidase-derived superoxide (O_2_˙) [13] production as well as upregulation of major histocompatibility complex (MHC)-II antigens [14].

The recruitment and activation of various T cell subsets in several forms of human and experimental GN has also been well characterized. The presence of Th1 and Th2 subsets of primed CD4^+^ T helper cells have been identified and reported to be associated with different patterns and severities of glomerular injury. Th1-predominant responses are associated strongly with aggressive forms of GN, such as rapidly progressive (crescentic) GN, while Th2 responses are associated with non-aggressive forms, such as membranous nephropathy (MN) [8]. Th1 and Th2 cells can be functionally distinguished by their cytokine profiles and their ability to generate different types of immune effector responses. Th1 cells produce interferon (IFN)-γ, interleukin (IL)-2, tumor necrosis factor (TNFα), and lymphotoxin α (previously known as TNF-β). They promote macrophage activation and delayed-type hypersensitivity (DTH) effector responses. They also assist B cells in the production of complement-fixing antibody isotypes which mediate opsonization and phagocytosis. Th2 cells produce IL-4, IL-5, IL-10 and IL-13, thus promoting the production of non-complement fixing IgG isotypes and IgE, and are important in allergic responses. Two other CD4^+^ T cell subsets, Th17 (IL-17 producing T cells) and regulatory T cells (Tregs), have also been identified in GN lesions. Th17 cells differ from Th1 and Th2 cells in their cytokine expression profiles and cytokine synthesis which drive their differentiation. Th17 cells cause inflammation via the production of TNF-α, IL-17A, IL-17F, IL-21 and IL-22, leading to increased expression of numerous proinflammatory cytokines by intrinsic glomerular cells (endothelial, mesangial, and podocytes) and by infiltrating inflammatory cells. However, the levels of IL-17-producing T-cells in inflammatory lesions are often low and usually found together, with a more prominent infiltrate of Th1 cells, suggesting that the combined action of pathogenic Th1 and Th17 cells might be responsible for establishing autoimmune forms of GN [9]. Tregs (CD4^+^CD25^+^ T cells) confer potent protection from immune-mediated renal injury by downregulating Th1 or Th17 effector T-cell responses via STAT3 activation, which induces a C-C motif chemokine receptor 6 (CCR6^+^)/Treg17 phenotype, uniquely equipped to suppress pathogenic Th17 responses in the course of acute and chronic GN [15].

Cytotoxic cells also play a role in the pathogenesis of GN. Cytotoxic CD8^+^ T cells are found predominantly in the periglomerular area, and their presence was shown to correlate with poor GN outcomes, while CD8^+^ T cell signatures also denote a poorer prognosis [16,17]. These cells can disrupt the continuity of the Bowman’s capsule [18] and can cause direct injury to intrinsic glomerular cells via the perforin/granzyme pathway [19]. In contrast to cytotoxic CD8^+^ T cells, which need priming by antigen-presenting cells (APCs) to exert cytotoxicity, natural killer (NK) cells can kill target cells without any priming or prior activation. Their presence has been documented in aggressive forms of GN, localized in the interstitium and peri-glomerular areas, and only rarely within glomeruli [20]. Finally, their exact role in the pathogenesis and outcomes of GN remains to be elucidated [21].

## 3. Effect of Mps on Effector Mechanisms of Renal Immune Injury

### 3.1. Mps, HO Activity and HO Isoform Expression

**Figure 1:**
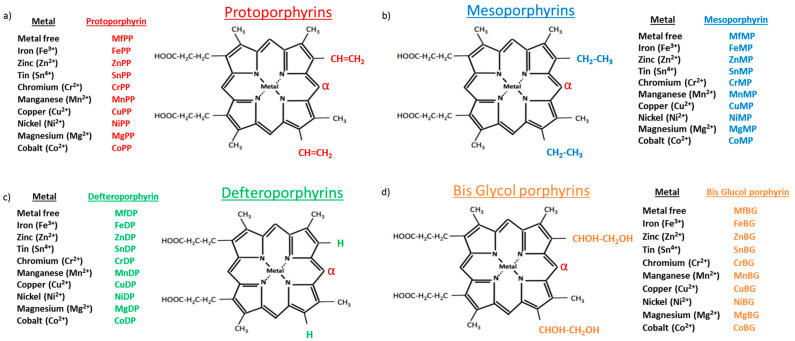
Metalloporphirn (Mps) types. Schematic representation of the chemical structure of Mp molecules depending on the ring substituents (lower right and upper right) and the central metal moiety. Porphyrins catalyzed by HO are oxidized at the α-position (red colour) to yield a tetrapyrrole. Modified from Vreman et al. (2001) [22].

Porphyrins are heterocyclic organic compounds composed of four pyrrole subunits interconnected via methene bridges. They are essential for the function of molecules such as chlorophylls and cytochromes and form complexes with redox-active transitional metals to generate Mps. Depending on the substitution of the side chain of the porphyrin ring, different types of Mps may arise, including protoporphyrins (PP), mesoporphyrins (MP), deuteroporphyrins (DP) and bis-glycol porphyrins (BG) (Figure 1). Furthermore, a wide range of each Mps type arises depending on the central metal moiety (Figure 1).

**Figure 2:**
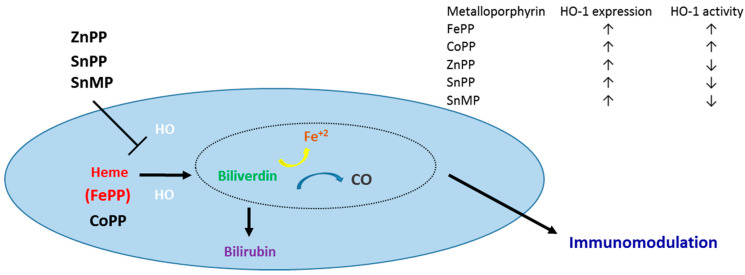
Effect of Mps on HO reaction and HO-1 expression and activity. Schematic representation of the HO reaction and the in vivo effect of various Mps which, depending on metal moiety, act either as HO activators/inducers (FePP and cobalt protoporphyrin, CoPP) or HO activity inhibitors (ZnPP, SnPP and tin mesoporphyrin, SnMP). Mps may also have a differential effect on HO-1 expression and activity in vivo.

Mps are well known inducers or inhibitors of the enzymatic activity of HO, the antioxidant, cytoprotective and immunoregulatory enzyme that catalyzes degradation of heme, also known as iron protoporphyrin (FePP), (Figure 2) to biliverdin, ferrous iron (Fe^+2^), and carbon monoxide (CO). HO-1 and HO-2 are the two major HO isoforms identified in mammalian cells. While HO-2 is constitutively expressed in basal levels in most tissues, HO-1 is the inducible HO isoform, whose expression is highly increased following exposure to different types of stress. HO-2 shares a high sequence homology and has a very similar structure with HO-1 [23,24,25], especially for regions around the catalytic site which FePP binds and is catabolized. In enzymatic analysis studies, V_max_ of HO-2 for FePP catalysis is approximately one-tenth of that of HO-1 while the K_m_ is three times higher. HO-2 has been viewed as the provider of baseline FePP degradation/detoxification activity in the absence of stress. HO-2 has not been studied as extensively as HO-1 and less information is available on its role in cytoprotection compared with HO-1.

The effect of various Mps on the enzymatic activity of HO isoforms has been investigated previously [26]. Studies determined the IC_50_ concentrations of Mps for HO-1 and HO-2 enzymatic activities inhibition, utilizing rat spleen and brain tissue samples, respectively, and an in vitro enzyme activity measurement method. For each Mp tested, HO-2 activity inhibition was similar or even greater than HO-1 inhibition. The order of Mp potency for activity inhibition was similar for both isoenzymes with greater inhibition observed for tin protoporphyrin (SnPP), albeit exhibiting greater selectivity for HO-2. PPs containing Zn as the redox-active metal moiety were the least effective in inhibiting HO activity, mainly attributed to HO-2 inhibition, while none of the Mps tested showed an exclusive HO-1 selectivity. These studies have provided valuable information for the correct selection of Mps, when needed for therapeutic strategies. This is because the preservation of HO activity, deriving from the HO-2 isoform, would be ideal or essential in order to maintain baseline levels of FePP degradation and cytoprotection.

The effects of Mps on HO enzyme activity and isoform expression can occur in tandem and can be discordant with different responses. This was convincingly demonstrated using zinc protoporphyrin (ZnPP), a naturally occurring Mp that potently increases HO-1 synthesis but inhibits HO enzyme activity. ZnPP was the strongest HO-1 inducer of all HO activity inhibitory Mps tested (ZnPP, SnPP, and chromium protoporphyrin (CrPP)) in cultured cells. The increase in HO-1 synthesis was independent of HO enzyme activity and was mediated via binding of the early growth response-1 (Egr-1) transcription factor to the consensus sequence on the HO-1 promoter [27]. These observations indicate that the ZnPP-mediated HO-1 induction may serve as a signaling mechanism in the cell cycle regulation. Further studies showing guanylate cyclase activity inhibition [28] and suppression of cell proliferation (mainly cyclin D) and angiogenesis gene expression by ZnPP, [29] via mechanisms independent of HO activity inhibition, confirmed the above observations.

Taken together, observations above strongly indicate that the central metal moiety determines unique properties of Mps regarding HO activity and isoform expression (Figure 2). FePP, CoPP, ZnPP, SnPp, and SnMP are the most extensively studied Mps with data available from both human and animal studies. Data on other Mps such as BGs and some DPs are mainly derived from studies testing their potential as agents for the treatment of hyperbilirubinemia. Reports have stressed the great HO inhibitory effect of ZnBG, MnBG and CrBG as well as of ZnDP and CrDP and the increased photoreactivity of ZnDP, SnDP and SnBG [22]. The effect of these Mps on HO-1 expression however was not assessed.

### 3.2. Role of Mps in Complement Mediated Renal Immune Injury

FePP concentrations are expected to increase within glomerular capillaries, particularly in aggressive forms of GN associated with hematuria or hemoglobinuria. This is because red blood cells passing through nephritic glomeruli undergo injury/lysis, resulting in the release of hemoglobin (Hb) and FePP. Although the concentrations of “free” FePP attained within glomeruli remain unknown, in various hemolytic diseases, such as the hemolytic uremic syndrome, FePP concentrations, ranging from 20 to 50 µM, have been reported [30,31]. At these concentrations, the levels of hemopexin (HPX), a plasma protein with the highest binding affinity for FePP, are depleted, and free FePP can exert cytotoxic effects. These include activation of the alternative complement pathway in plasma, the release of C3a, C5a and soluble C5b-9 (membrane attack complex, MAC) and the promotion of C3 and C5b-9 binding to FePP-exposed cells [32]. Effects of free FePP further include decreased expression of complement regulatory proteins (CRPs) that mitigate complement activation, membrane cofactor protein (MCP) and decay-accelerating factor (DAF) [32]. Moreover, FePP interacts with factor I, a serine protease that cleaves C3b, and this interaction interferes with factor I-mediated degradation of C3b leading to excessive C3-convertase formation and C3-cleavage potentially causing sustained amplification of the complement alternative pathway [33]. These adverse effects are mitigated, in part, by FePP degradation following HO-1 induction in intrinsic glomerular cells exposed to FePP or by FePP uptake by infiltrating leukocytes capable of endocytosing FePP:HPX complexes (see Section 3.3).

HO-1 induction has been demonstrated in all intrinsic glomerular cells (endothelial, mesangial, and podocytes) following exposure to FePP in vitro. Of particular interest are podocytes, which are most vulnerable to immune-mediated injury due to their terminally differentiated nature. Furthermore, podocyte expression of DAF was found to be induced by FePP via an HO-1-dependent mechanism. In rat glomeruli, constitutive DAF expression has been demonstrated exclusively in podocytes [34]. Thus, incubation of isolated rat glomeruli with FePP, at concentrations likely to be found in the circulation of patients with hemolytic disorders, increased HO-1 and DAF expression, with both proteins immunolocalized in podocytes [35]. This effect was attenuated in glomeruli from HO-1-deficient rats and augmented in glomeruli from rats with podocyte-targeted HO-1 overexpression. To determine the role of the metal or porphyrin moiety of FePP in causing DAF induction, glomeruli were incubated with CoPP, ZnPP, SnPP, SnMP, or with the metal-devoid porphyrin (PPIX). It was shown that PPIX alone at high concentrations was a weak DAF inducer, while SnPP was the most potent [36]. As mentioned earlier, these non-Fe Mps cannot be oxidatively degraded by HO because, in contrast to FePP, they have no oxygen-binding capacity. Similar to FePP, CoPP induces HO-1 (mRNA and protein) in vitro even though it inhibits HO enzyme activity. However, it increases both HO-1 protein synthesis and HO enzyme activity in vivo due to its strong activation of HO-1 gene expression [37]. SnPP markedly increases HO-1 synthesis both in vitro and in vivo but potently inhibits HO enzyme activity [38]. Similarly, ZnPP potently induces HO-1 expression but inhibits HO activity [27]. SnMP reduces both HO-1 expression and HO activity [39]. All these non-Fe Mps are competitive inhibitors of HO enzyme activity, having a much higher binding affinity to HO than FePP. Taken together, these observations indicate that the metal moiety of Mps determines the potency of DAF induction in glomeruli and that this effect was independent of HO enzyme activity.

### 3.3. Role of Mps in Leukocyte Mediated Renal Immune Injury

The metal and porphyrin moieties of Mps play separate roles in determining processes (reviewed above) relevant to the pathogenesis of renal immune injury. In vitro studies using human and mouse peripheral blood mononuclear cells (PBMCs) which infiltrate the kidneys in various forms of GN and IN, demonstrated that the porphyrin moiety of FePP, PPIX, which is a precursor of FePP but not a HO-1 inducer, is necessary and sufficient to suppress the signal transducer and activator of transcription 1 (STAT1) protein (the primary transcription factor activated by interferons) expression and activity and to attenuate STAT1-dependent immune responses including interferon regulatory factor 1 (IRF1) and interferon-induced protein 10 (CXCL10) expressions [40]. In the same cells, FePP and SnPP were found to be mitogenic, while the non-mitogenic metal-devoid PPIX could be rendered mitogenic by the chemical insertion of iron [41]. Mitogenicity of both Mps in PBMCs was markedly enhanced by low concentrations of IL-2. Furthermore, these Mps, in combination with IL-2, increased IL-2R on PBMCs. Scavengers of oxygen-free radicals inhibited FePP-induced mitogenicity FePP but not SnPP-induced mitogenicity, indicating that an oxidative event is needed to mediate the mitogenic effect of the former Mp. FePP alone and, to a greater extent, in combination with IL-2, enhanced PBMCs cytotoxicity. In contrast, SnPP, a more potent mitogen than FePP, had a marked inhibitory effect on cytotoxicity induced by IL-2. Both Mps stimulated TNF-α and IFN-γ production by PBMCs, and IL-2 acted synergistically in eliciting this effect [42]. These observations are of relevance when considering the use of FePP or SnPP as disease-modifying agents in renal immune injury in which infiltration by activated PBMCs, IL-2 production and intraglomerular oxidative stress are prominent features.

In T cell subsets that play a role in renal immune injury (see above), FePP-mediated HO-1 induction regulates effector responses as shown in Th1, Th2 and Treg cells. HO-1 induction inhibits activation and proliferation of activated CD4^+^ T cells apparently via the production of the FePP degradation metabolites, biliverdin and CO, which suppress T cell proliferation by inhibiting IL-2 production [43]. Furthermore, HO-1 induction was shown to promote the generation of Th2 cytokines such as IL-4 and IL-10 and to suppress the secretion of the Th1 cytokine IFN-γ thereby shifting the balance of the Th1/Th2 response from immune injury towards Th2 predominance [44]. In Th17 cells, which primarily produce the IL-17 cytokine, HO-1 induction by FePP blocks their differentiation partly by suppressing IL-6. This effect is apparently mediated by the FePP degradation metabolite, CO, which inhibits the production of IL-6, a key cytokine priming Th17 cell differentiation and a downstream mediator of IL-17 effects [45].

In Tregs (CD4^+^CD25^+^), which play a significant role in the induction of immune tolerance and protection from immune-mediated renal injury by downregulation of Th1 or Th17 T effector cell responses, constitutive HO-1 expression is higher than in CD4^+^CD25^-^ T cells [46]. The forkhead box P3 (Foxp3) transcription factor, which is crucial for the development of Tregs [47], was shown to induce HO-1 [48], while inhibition of Treg HO-1 activity using SnPP inhibits their development [49], indicating that the use of this Mp in renal immune injury would be detrimental. This was demonstrated in a model of experimental membranous glomerulopathy [50], in which CD4^+^ and CD8^+^ effector T cells were required for induction of injury [51,52].

Lastly, HO-1 induction by CoPP inhibits MHC class II expression in APCs and attenuates cytotoxic CD8^+^ T cell accumulation, proliferation, and effector function in autoimmune inflammation via a mechanism involving APCs (mainly dendritic cells, DC). This effect was mediated, in part, by the FePP degradation metabolite, CO [53]. In contrast, HO-1 depletion by small interfering RNA (siRNA) in DCs results in MHC class II upregulation and a DC-directed T cell response preferentially toward a CD4^+^ T cell rather than a CD8^+^ T-cell response [54]. The role of HO-1 induction by Mps in cytotoxic CD8^+^ T cell and NK-mediated renal immune injury has yet to be elucidated. Of note, however, in a mouse model of lupus nephritis, treatment with 5-aminolevulinic acid, a key progenitor of FePP synthesis, increased the number of Tregs and reduced the population of activated DCs and CD8^+^ T cells at an early stage of the disease [55].

The translational relevance of in vitro studies cited above is unknown because concentrations of FePP or non-Fe Mps used may not reflect those attained in cells or in circulation. FePP concentrations in the human body are maintained at extremely low levels owing to FePP scavenging proteins (HPX, albumin, α1-microglobulin and α1-antitrypsin) and to HO-1 induction that degrades FePP. For example, in a patient with an inherited HO-1 deficiency, a FePP concentration of ~0.5 mM was found and was associated with generalized oxidative tissue injury, organ failure and death [56]. Furthermore, based on the Km of the HO-1 reaction (1 µM), the free FePP concentration in normal cells is likely below 1 µM. Depending on which method is used to measure free FePP, its concentration in erythrocytes ranges from 0.1 to 0.15 µM, whereas it increases three- to five-fold in patients with hemolytic disorders such as sickle cell anemia [57]. FePP concentrations above 1 µM are considered high, and it is mainly at these concentrations that FePP is degraded by HO to biliverdin and CO. FePP concentrations above 10 µM are found only in inherited HO-1 deficiency.

As mentioned earlier, intrarenal concentrations of free FePP following immune injury resulting in hematuria/hemoglobinuria remain unknown, as well as whether these FePP concentrations induce HO-1 or other cytoprotective molecules. Based on experimental and human models of renal immune injury, it can be inferred that FePP is generated at concentrations sufficient to induce HO-1 in leukocytes infiltrating glomeruli [58], in tubular epithelial cells [59] and in the cells present in urine sediment [60]. However, HO-1 inducers other than free FePP could also increase HO-1 expression in these cells. To determine whether free (unbound) FePP in serum is sufficient to induce CRPs, isolated rat glomeruli were incubated in media supplemented with HPX-deficient (HPX^−^) or HPX-containing (HPX^+^) sera as a means of achieving different concentrations of unbound FePP and its partitioning between media and glomerular cells. Expression levels of HO-1, DAF and CD59 proteins increased in the glomeruli incubated with HPX^−^ sera compared to levels in glomeruli incubated with HPX^+^ sera, suggesting that increased unbound FePP was present in sufficient concentrations to increase expression of HO-1 and those CRPs [60].

Uptake of free FePP by leukocytes infiltrating kidneys following immune injury and capable of endocytosing FePP:HPX complexes is an additional mechanism whereby HO-1 can mitigate the extent of the injury. These cells are primarily infiltrating macrophages. As mentioned above, erythrocytes undergoing damage/lysis within glomerular capillaries in the course of aggressive forms of GN can be a source of free Hb and FePP. Both rapidly form complexes with the scavenging plasma proteins, haptoglobin (Hp) and HPX [61]. Glomerular filtration of these complexes is impaired because of their size [62], thus facilitating delivery to infiltrating macrophages and internalization by specific macrophage receptors. Hb:Hp complexes are internalized by the CD163 receptor while FePP:HPX complexes by the low-density lipoprotein receptor-related protein, also known as CD91 receptor [63]. Internalized Hb:Hp or FePP:HPX complexes were shown to induce HO-1 [64], thus creating an intraglomerular HO-1 overexpressing macrophage population with an anti-inflammatory/repair promoting phenotype (traditionally referred to as M2) [65]. M2 macrophages were shown to attenuate injury in GN [66].

## 4. Use of Mps as Disease Modifying Agents in Renal Immune Injury

Both Fe- and non-Fe Mps have been viewed as therapeutic agents in situations where mitigation of oxidative/nitrosative stress is desired as MP side-chain substitutions tune MP redox properties to act as potent superoxide dismutase mimics and peroxynitrite decomposition catalysts [67]. Furthermore, Mps capable of inducing HO-1 (FePP, CoPP) have been viewed as therapeutic agents in situations where HO-1 induction is desired. Consideration of HO-1 induction is based on established broad anti-inflammatory and immunomodulatory effects of FePP degradation by HO-1 to cytoprotective metabolites (mainly biliverdin and CO) [43] and on positive effects on maturation of myeloid cells and their mobilization from the bone marrow to peripheral blood [68]. Both FePP and non-Fe Mps have been used in clinical practice or clinical trials. For example, FePP (also known as hematin) remains the only specific treatment and a first-line drug in the treatment of acute porphyria [69], while other non-Fe Mps have been proposed for the treatment of disorders, including ischemic injury [70], neonatal hyperbilirubinemia [71], and neutropenia [68]. Furthermore, the metal-devoid PPIX, which is the immediate biosynthetic precursor of FePP, has been used in photodynamic therapy of specific skin disorders and tumors [72]. Earlier studies on the clinical use of Mps as disease-modifying agents focused on those that competitively inhibit the total HO enzyme activity (ZnPP, SnPP, MnPP), thereby reducing bilirubin production in diseases associated with hyperbilirubinemia [73], primary biliary cirrhosis, hemochromatosis [74], and porphyria [75]. HO inhibitory Mps that were comprehensively evaluated (efficacy and safety) include SnPP, ZnPP, zinc mesoporhyrin (ZnMP), and zinc deuteroporphyrin bis glycol (ZnBG). Side effects reported include photosensitization/phototoxicity, long-term tissue deposition, HO inhibition (due to the inability for their oxidative degradation by HO) and effects on hematopoiesis, including iron deficiency.

In animal models of renal immune/inflammatory injury, HO inhibitory Mps have been used to provide evidence that preservation of HO activity is important. However, it should be emphasized that these Mps inhibit HO activity originating both from HO-1 (the inducible HO isoform) and HO-2 (the constitutive isoform). Because the catalytic activity of HO from either isoform elevates cellular iron content, a potent facilitator of oxidative stress, interpretation of the results from studies using HO inhibitory Mps should also take into consideration iron-dependent effects on the outcomes of the injury. This was convincingly shown in an in vivo model of chronic tubulointerstitial nephropathy developing in hemolytic diseases and attributed to the long-term exposure of renal tubular cells to FePP. This study demonstrated an increased expression of monocyte chemoattractant protein (MCP)-1, as well as HO-1, in renal proximal tubular cells (RPTC), following exposure to FePP with a simultaneous increase in levels of catalytically active intracellular iron. A subsequent in vitro study revealed that MCP-1 induction was HO activity-dependent as the HO activity inhibitor, ZnPP, blocked HO activity, attenuated the rise in catalytically active iron, and prevented the increase in MCP-1 expression with no effect on FePP-induced HO-1 mRNA levels. The authors concluded that exposure of RPTCs to FePP induces HO-1, but it is probably the attendant increase in HO activity that elicits MCP-1 induction, possibly via increased catalytically active intracellular iron. Thus, an iron chelator blocked FePP-induced upregulation of both HO-1 and MCP-1 genes, whereas a cell-permeant form of iron directly induced these genes [76,77].

In a murine experimental MN model by intravenous injections of cationic bovine serum albumin resulting in proteinuria, HO-1 expression levels in cortical homogenates remained similar to those of the control group. Intraperitoneal administration of CoPP augmented HO-1 protein expression, which immunolocalized in glomeruli and tubules, while SnPP had no significant effect. CoPP treatment was shown to reduce proteinuria, attenuated glomerular immune complex deposition and severity of glomerular lesions, lipid peroxidation, and apoptosis. Moreover, it decreased the expression of proinflammatory cytokines in the renal cortex and increased the expression of the anti-inflammatory cytokine IL-10. Similar effects were not observed by the administration of SnPP apart from a reduction in cortical proinflammatory cytokine production, albeit without an IL-10 increase. As mentioned earlier, CoPP increases both HO-1 protein synthesis and total HO enzyme activity in vivo [78]. In contrast, SnPP markedly increases HO-1 synthesis in vivo but potently inhibits HO activity in vitro. Therefore, the differential effect of Mps on the total HO activity rather than their ability to induce the HO-1 isoform should always be taken under serious consideration.

The HO reaction is a key, if not the sole, mechanism by which cells can generate carbon monoxide (CO) originating from FePP degradation. To determine whether CO can mimic beneficial outcomes of Mps used to increase total HO enzyme activity in renal immune injury, mice with renal immune injury due to lupus nephritis were exposed to compressed CO at a concentration of 250 parts per million (ppm) for 2 h per day for up to 30 weeks. CO treatment significantly decreased infiltration by activated B220^+^ CD4^−^ CD8^−^ T cells in kidneys. Moreover, it decreased the extent of glomerular proliferative lesions and proinflammatory cytokine production and delayed the decline in kidney function [79]. An additional mechanism by which CO can mediate anti-inflammatory effects is the inhibition of Toll-like receptor (TLR) 2, 4, 5, and 9 signaling in infiltrating macrophages [80], which, as mentioned above, play a key role in the pathogenesis and progression of renal immune injury.

Of the various naturally occurring Mps, FePP is the most extensively used as a disease-modifying agent in renal immune injury. Reasons include: (1) it is the natural substrate/inducer of HO by which it is oxidatively degraded to cytoprotective metabolites (biliverdin, CO), (2) it is approved for clinical use (in the form of FePP arginate, Normosang) in patients with an enzymatic block in FePP biosynthesis (intermittent acute porphyria, porphyria variegata, and hereditary coproporphyria), and (3) it is well tolerated when administered in high doses (3 mg/kg intravenously) without any significant changes in coagulation and fibrinolysis parameters in healthy volunteers other than a transient decrease in coagulation factors IX and X [81].

Early studies demonstrated that in an aggressive form of experimental GN in rats, FePP (30 mmol/kg subcutaneous administration prior to and 3 days following induction of GN) markedly reduced proteinuria, neutrophil and macrophage infiltration, and extent of intraglomerular thrombus formation. Of note, HO-1 was not immunohistochemically detected in the normal glomeruli but was found to be induced in infiltrating macrophages following the onset of GN. It was proposed that FePP-mediated HO-1 induction attenuated the severity of GN via the production of bilirubin and CO [58] (Table 1). Additional studies in the same model confirmed HO-1 induction in infiltrating macrophages and demonstrated that iNOS expression and enzyme activity in these cells also increased. Administration of subcutaneous FePP treatment prior to the induction of GN increased glomerular infiltration by HO-1 overexpressing macrophages at early stages (24 h following induction) and reduced proteinuria as well as iNOS expression and activity. It was suggested that HO-1 negatively regulates iNOS in infiltrating macrophages, indicating a switch from a pro- to an anti-inflammatory phenotype [82]. Interestingly, iNOS-derived NO was a negative regulator of iNOS and also suppressed cyclooxygenase (COX)-2 levels but maintained HO-1 levels [76]. Long-term subcutaneous FePP administration (initiated 24 h before induction of GN and subsequently administered on days 1, 3, and 6 thereafter to achieve sustained HO-1 induction) increased HO-1 expression (assessed by immunolocalization) in infiltrating macrophages and tubular epithelial cells. This was associated with a sustained reduction in proteinuria (up to 14 days) following the onset of GN in FePP-treated animals, in which blood urea nitrogen (BUN) also decreased without a change in serum creatinine or systemic blood pressure [77].

These studies established the sustained beneficial effect of FePP treatment on proteinuria in an aggressive form of GN and implicated HO-1 induction in infiltrating macrophages as a potential mechanism. Although there are no studies comparing the efficacy of routes of FePP administration on outcomes of glomerular immune injury, the dermal route may be of advantage. In a study assessing the inhibitory effect of the intradermal administration of HO-1 inducers on effector T cells sensitized by specific antigen in mice, it was demonstrated that CoPP inhibited antigen-specific effector T cells when injected intradermally together with the T-cell cognate antigen. The intradermal injection resulted in the recruitment of HO-1^+^ monocyte-derived DCs exclusively to lymph nodes draining the site of injection. Following an encounter with HO-1^+^ monocyte-derived DCs, the ability of effector T cells to migrate towards chemokine gradients was reduced, resulting in impaired accumulation to the inflamed organ. This phenomenon was observed both in a CD8^+^ T cell-mediated injury model of type 1 diabetes mellitus and in a CD4^+^ T cell-dependent injury model of multiple sclerosis. Further, intradermal co-injection of the clinically approved HO-1 inducer, FePP arginate, (Normosang), with a specific antigen to non-human primates resulted in the accumulation of HO-1^+^ monocyte-derived DCs in lymph nodes draining the site of injection and the suppression of delayed-type hypersensitivity reaction [86]. Although not confirmed in models of renal immune injury, these observations justify the intradermal administration as the preferred route of HO-1 inducers as both CD4^+^ and CD8^+^ cells have been implicated as effectors of injury (see above).

The demonstration that FePP treatment induces HO-1 in macrophages infiltrating glomeruli following the onset of immune injury raised the question of whether HO-1 induction in intrinsic glomerular cells has a similar effect. To address this question, mice with visceral glomerular epithelial cell (podocyte)-targeted HO-1 overexpression were generated using a mouse nephrin promoter [87]. Induction of an aggressive form of glomerular immune injury following administration of an antibody against the glomerular membrane (anti-GBM) in mice with podocyte-targeted HO-1 overexpression resulted in proteinuria that was significantly lower compared with that in wild-type (WT) mice on days 3 and 6 following administration of this antibody [88]. As podocytes are a key barrier in glomerular capillary permeability to protein, the reduction in proteinuria observed in these studies indicates that HO-1 overexpression in podocytes maintained the integrity of their barrier function. This effect apparently involved the preservation of podocyte nephrin expression [89], which is a key structural component of the permeability barrier.

The significance of preserving podocyte structural/functional integrity by HO-1 becomes apparent when considering earlier observations that, compared with other nephron segments, the ability of intrinsic glomerular cells, including podocytes, to upregulate HO-1 expression is limited. Thus, systemic (intravenous) administration of established HO-1 inducers, including Hb or FePP, causes a robust HO-1 induction in tubules but not in glomeruli [90,91]. Induction in tubules following Hb administration required its filtration and luminal uptake as the reduction in Hb filtration by chemical modification to stabilize its presence in plasma significantly attenuated tubular HO-1 induction but failed to induce it in glomeruli [90]. Similarly, immune injury using an antibody directed against the glomerular basement membrane failed to cause an appreciable HO-1 induction in intrinsic glomerular cells despite the release of potent HO-1 inducers (i.e., O^−^-derived oxidant radicals and cytokines). Likewise, non-immune injury directed against podocytes and also associated with the release of potent HO-1 inducers failed to induce HO-1 in these cells [91,92]. However, a long-term (six months) follow-up of rats with podocyte-targeted HO-1 overexpression in which no injury was induced had detrimental effects, including proteinuria and glomerular scarring [93]. Taken together, these observations raised the question of whether HO-1 expression in podocytes is subject to “tight” control. To address this question, glomeruli were isolated from rats with podocyte-targeted HO-1 overexpression generated using Sleeping Beauty (SB) transposon-mediated transgenesis or from WT controls and incubated for defined time periods with increasing FePP or CoPP concentrations. These Mps dose-dependently increased HO-1 protein, but this increase was not sustainable as HO-1 protein levels returned to basal after reaching a “threshold” induction level following long-term (12 h) exposure to high FePP or CoPP concentrations. In glomeruli with podocyte-targeted HO-1 overexpression, the HO-1 induction threshold was reached at significantly lower FePP or CoPP concentrations [36]. These observations support the presence of a HO-1 expression control mechanism in glomeruli, particularly in podocytes, that may serve to prevent adverse consequences resulting from sustained HO-1 induction.

Taken together, the observations reviewed above indicate that while HO-1 overexpression in infiltrating macrophages is associated with the attenuation of glomerular immune injury, a similar outcome cannot be expected following HO-1 overexpression targeted to podocytes, as the long-term overexpression in these cells may have detrimental effects on its own (in the absence of on-going injury) [93]. Therefore, the former approach can be expected to have better outcomes. Observations from studies that employed adoptive transfer of engineered macrophages support this argument. Injection of bone marrow-derived macrophages (BMDM) with adenovirus-transduced IL-10 overexpression in the renal artery of rats with anti-GBM-mediated GN resulted in efficient localization in glomeruli and markedly reduced albuminuria compared to rats with GN receiving injections of non-transduced BMDM. The number of glomerular ED1- and ED3-positive macrophages, the extent of MHC class II expression, and the number of fibrinoid lesions were also reduced [94]. Studies using adoptive transfer of HO-1-overexpressing macrophages in models of renal immune injury have not been performed. However, because HO-1 induction in macrophages promotes the development of an anti-inflammatory (traditionally known as M2) phenotype [95], such studies are likely to show outcomes similar to those observed using the adoptive transfer of IL-10 overexpressing macrophages (see above). Thus, HO-1-derived CO, as expected to occur in HO-1-overexpressing macrophages, was shown to upregulate IL-10 and to polarize macrophages toward an M2 phenotype by increasing the expression of genes involved in M2 polarization [83].

In lupus erythematosus-associated nephritis, FePP treatment was also shown to have favorable outcomes. In lupus-prone mice, FePP levels in serum, kidney, and spleen lymphocytes negatively correlated with levels of proteinuria. Moreover, FePP supplementation at 15 mg/kg significantly ameliorated the syndrome of lupus, extended lifespan, reduced proteinuria, and alleviated splenomegaly and lymphadenopathy [84]. In the same model of lupus-prone mice, intraperitoneal injection with 100 μmol/kg FePP once a week beginning at 6 weeks of age to 21–24 weeks significantly reduced proteinuria and severity of glomerular lesions and decreased immune deposits in glomeruli. In addition, circulating IgG anti-double-stranded DNA antibody levels were significantly decreased in FePP-treated mice when compared with the controls. Furthermore, a single intraperitoneal injection of FePP reduced iNOS expression levels in the kidney and spleen, as well as serum IFN-γ levels. The authors concluded that HO-1 induction achieved by FePP treatment attenuated the severity of lupus nephritis by suppressing NO-dependent inflammatory responses and by reducing the production of pathogenic autoantibodies [85]. In patients with lupus nephritis, HO-1 levels were found to be decreased in all subsets of monocytes and activated neutrophils compared with healthy controls. HO-1 levels in the renal tissue of these patients were also lower, as were HO-1 levels in infiltrating immune cells [92]. The significance of HO-1 deficiency in the renal tissue and immune cells in patients with lupus remains unknown but could be detrimental given the beneficial outcomes of FePP supplementation described above. In the same disease, HO-1 expression was found to be defective in the majority of glomerular M2-like macrophages of patients studied [96].

## 5. Mps Toxicity Considerations

It can be surmised from reviewing the use of Mps as modifying agents in immune-mediated kidney injury that FePP and CoPP are the most promising in terms of favorable outcomes. FePP (available as FePP arginate-Normosang-formulation in Europe and South Africa and as FePP hydroxide-panhematin—in the United States) was first used in the early 1970s and has been commercially available since 1983. FePP arginate infused as a single intravenous dose of 3 mg/kg body weight to healthy volunteers was well tolerated. This dose and route of administration resulted in maximal plasma FePP concentrations of 51.5 ± 10.0 µg/mL, reached in 30.0 ± 21.2 min, and was sufficient to significantly decrease plasma HPX concentrations. The elimination half-life for FePP was 11.0 ± 2.2 h. All parameters assessed (platelet count, coagulation cascade factor levels, and fibrin degradation products) remained stable except for a transient but insignificant decrease in Factor X at maximum plasma FePP concentration [81]. In patients with acute intermittent porphyria, a typical FePP treatment regimen is 3 to 4 mg/kg body weight given intravenously as a single daily dose for four days or longer. Excessive dosing can cause reversible kidney injury (acute tubular necrosis) and, rarely, liver failure. The fate of Fe present in exogenously administered FePP is recycling and use for erythropoiesis [97]. However, iron overload can develop after multiple FePP treatments.

In clinical studies using non-Fe Mps (mainly SnPP and SnMP), the main goal was the inhibition of HO enzyme activity and bilirubin production. These studies (reviewed in Schulz et al.) were performed to assess efficacy, safety, and outcomes in patients with various hyperbilirubinemic syndromes treated with these Mps. Side effects reported include photosensitivity manifested as erythema [98] and iron deficiency anemia [99]. There are no reports of clinical trials using CoPP. However, its inorganic trace metal, cobalt (Co), has been used in the past (CoCl_2_, 25–50 mg orally daily for 12 weeks) to promote erythropoiesis in patients with anemia due to chronic kidney disease [100]. Side effects reported from long-term Co exposure include cardiomyopathy [101], hypothyroidism with goiter [102], and neurotoxicity [103]. A pharmacokinetic comparison between the trace element, Co, and its protoporphyrin chelate, CoPP, following subcutaneous administration of CoCl_2_ or CoPP in rats showed that Co was found predominantly (>95%) in plasma, from which it was rapidly eliminated (t (1/2) ≃25 h). CoPP was also found almost exclusively in plasma (>95%) but had a much slower elimination rate (≃3 days) compared with the non-complexed metal. Four weeks after dosing with either, the kidney retained the highest levels of Co, although, in CoPP-treated rats, elevated levels of Co were still present in the spleen, gonads, lung, and thymus [104].

Toxicity consequent to the accumulation of the PPIX moiety of Mps following long-term treatment should also be considered. PPIX is hydrophobic and deposited in lipid layers of cell membranes. It is photoreactive and absorbs light causing the protoporphyrin to enter an excited energy state. Energy transfer to dissolved oxygen forms the superoxide ion (O_2_^−^), which in turn can generate hydroxyl ions (OH^−^). These highly oxidizing species of oxygen can interact with many biological molecules, such as proteins, lipids, and DNA, and can form adducts with carbon-carbon double bonds [105]. Skin and liver injury are the most frequent adverse consequences reported.

## 6. Concluding Remarks

There is solid evidence that Mps can modulate (improve or worsen) the severity of a renal immune injury, and a plethora of preclinical (animal) studies strongly support their role as disease-modifying agents. To this end, the ability of an MP to increase HO enzyme activity rather than to primarily induce the HO-1 isoform in vivo as well as the ability of HO to oxidatively degrade the MP employed, should be carefully considered. Only Mps that are oxidatively degradable by HO, such as FePP, can generate the cytoprotective metabolites biliverdin, bilirubin, and CO. Certain non-degradable Mps, exemplified by CoPP, can cause a sustained increase in HO-1 induction and HO activity in vivo with consequent degradation of intracellular FePP. This may result in FePP depletion to levels that may impair the function of FePP-containing enzymes, such as NADPH, regulating important cell functions. Other non-degradable Mps, exemplified by SnPP and ZnPP, markedly induce HO-1 synthesis in vivo but potently inhibit total HO enzyme activity, and their administration has detrimental effects on renal function under conditions of injury.

FePP is the prototypic HO-degradable Mp and the only one approved for clinical use. Its efficacy in preserving renal function in patients with immune-mediated injury is unknown. In aggressive forms of experimentally induced renal immune injury, as exemplified by aggressive forms of GN, HO-1-inducing Mps do so primarily in infiltrating leukocytes, particularly macrophages, rather than in intrinsic glomerular cells. Targeted HO-1 induction in the latter can be detrimental, as shown in podocytes. Therefore, HO-1 overexpression in the former could be a preferred strategy. The question of whether HO-1 overexpression in infiltrating leukocytes following systemic administration of HO-1-inducing Mps is a necessary and sufficient requirement to attenuate renal immune injury and preserve renal function remains unanswered as these Mps can have immunoregulatory effects independent of HO-1 induction.

## Figures and Tables

**Table 1:** Mp effects on immune-mediated kidney injury in animal disease models.

Study	Summary of Findings	Reference Number
Mosley K. et.al., 1998	Hemin treatment in a rat nephrotoxic nephritis model ameliorates disease via induction of HO-1 and reduction in glomerular macrophage infiltration	[58]
Data P.K et.al., 1999	Hemin treatment in a rat model of anti-glomerular basement membrane antibody-mediated glomerulonephritis reduces proteinuria via HO-1 induction and attenuation of iNOs expression.	[82]
Datta P.K. et.al., 2006	Long term hemin treatment attenuates proteinuria in a rat model of anti-glomerular basement membrane antibody-mediated glomerulonephritis.	[77]
Kang I.-S. et.al., 2021	Hemin treatment increased macrophage differentiation whereas ZnPP reduced macrophage differentiation in CORM mediated macrophage differentiation.	[83]
Wu B. et.al., 2021	Hemin treatment ameliorated lupus disease in lupus prone mice.	[84]
Takeda Y. et.al., 2004	Hemin treatment suppressed lupus nephritis via HO-1 induction and reduction in iNOs synthesis in lupus mice.	[85]

## Data Availability

Not applicable.
